# Natural killer cells and regulatory T cells in aneurysmal subarachnoid hemorrhage in peripheral blood and cerebrospinal fluid - a pilot study

**DOI:** 10.1186/s12987-026-00824-3

**Published:** 2026-05-29

**Authors:** A. Pfnür, A. Bohnacker, L. Dörfer, A. Ziebart, R. Halbgebauer, M. Huber-Lang, H. Tumani, C. R. Wirtz, T. Kapapa

**Affiliations:** 1 Department of Neurosurgery, University Ulm, Albert-Einstein-Allee 23, 89081 Ulm, Germany; 2 Institute of Clinical and Experimental Trauma Immunology, University Ulm, Helmholtzstraße 8/1, 89081 Ulm, Germany; 3 Department of Neurosurgery, Medical University of Innsbruck, Anichstraße 35, Innsbruck, 6020 Austria; 4 Department of Neurology, University Ulm, Oberer Eselsberg 45, 89081 Ulm, Germany

## Abstract

**Background:**

Delayed cerebral ischemia (DCI) after aneurysmal subarachnoid hemorrhage (SAH) is incompletely understood and may involve intrathecal immune activation. Cytotoxic natural killer (NK) cells can rapidly respond to SAH and promote neuroinflammation, whereas regulatory T cells (Tregs) may counteract excessive immune activation. This study investigates the NK/Treg ratio in cerebrospinal fluid (CSF) and peripheral blood (PB) as a potential immunological marker associated with DCI.

**Methods:**

In this prospective, observational, single-center study, CSF and peripheral blood samples from 21 patients with aneurysmal SAH admitted between 2023 and 2024 were analyzed. Longitudinal dynamics of NK cells and Tregs were assessed by flow cytometry at predefined time points from day 1 to day 14 after hemorrhage. The NK/Treg ratio was calculated for each compartment and time point, and its association with DCI was explored.

**Results:**

Twenty-one patients were included, of whom 16 were female (76.2%). The mean age was 55.9 years (standard deviation [SD] 13.3). Median WFNS and modified Fisher grades at admission were 3 (range 1–5) and 3 (range 1–4), respectively. DCI occurred in 10 patients (47.6%), with a median onset of 8.5 days after hemorrhage (range 4–12). Longitudinal dynamics of NK cells and Tregs in CSF and peripheral blood were assessed using flow cytometry. On day 3, the CSF NK/Treg ratio was higher in patients who later developed DCI compared with patients without DCI (mean 5556.6 [SD 12266.0] vs. 161.8 [SD 195.2]). CSF ratios remained numerically higher at later time points, whereas changes in peripheral blood were modest. In exploratory analyses of clinical covariates, lower modified Fisher grades (1–3 vs. 4) were associated with increased NK/Treg ratios in CSF at day 1 (*p* = 0.046) and day 2 (*p* = 0.007), possibly indicating that the elevation was not merely a reflection of subarachnoid blood burden.

**Conclusions:**

Patients who developed DCI showed an early intrathecal shift in the balance between NK cells and Tregs, reflected by a higher CSF NK/Treg ratio around day 3. This pattern was more pronounced in CSF than in peripheral blood, suggesting a compartment-specific immune alteration associated with DCI. Although the small sample size, high variability, and limited statistically significant findings preclude definitive conclusions, these data support the hypothesis that early CSF immune dysregulation may contribute to DCI after SAH. CSF-based immune profiling, including the NK/Treg ratio, may therefore represent a promising exploratory approach for DCI risk stratification. Larger prospective multicenter studies with standardized CSF sampling are required to validate these exploratory findings.

**Supplementary Information:**

The online version contains supplementary material available at 10.1186/s12987-026-00824-3.

## Introduction

Aneurysmal subarachnoid hemorrhage (SAH) is a life-threatening condition with high mortality and substantial long-term neurological disability [1]. A key contributor to poor outcome is delayed cerebral ischemia (DCI), which can lead to irreversible infarction days after the initial bleed [2]. Although angiographic vasospasm occurs in up to 50–67% of patients, only about one-quarter develop clinical DCI, indicating that vasospasm alone does not explain secondary injury [3]. DCI is now understood as a multifactorial process involving vasospasm, microvascular dysfunction, cortical spreading depolarizations and inflammation [4]. Consistent with this view, treating vasospasm alone has not eliminated DCI in clinical trials, suggesting that other mechanisms, particularly inflammation, play a critical role in secondary brain injury after SAH [5, 6]. An early inflammatory response begins within hours after SAH, marked by leukocyte influx into the CSF and subsequent activation of microglia, astrocytes, and adaptive immune cells [7].

The resulting cytokine surge disrupts the microvasculature and blood–brain barrier [8]. Higher inflammatory responses are associated with increased risk of DCI and worse outcomes [9]. This has spurred interest in deciphering the immune pathways involved in post-SAH complications such as vasospasm and DCI. Existing research emphasized neutrophils and monocytes in the CSF as potentially involved in the severity of vasospasm [10] or DCI [11] respectively. T cell dynamics seem to play a role in post-SAH complications [12]. In inflammatory stroke research, natural killer cells (NK cells) and regulatory T cells (Tregs) play opposing roles. NK cells contribute to early neuroinflammatory injury by releasing cytotoxic mediators and amplifying blood–brain barrier disruption [13], whereas Tregs exert neuroprotective effects by suppressing excessive immune activation and promoting resolution of inflammation, but Tregs seem to have adverse effects on the ischemic brain as well [14]. While T cells and NK cells are known to infiltrate or become activated in other CNS injuries, their specific roles in SAH remain incompletely defined. Analyzes of lymphocyte dynamics in SAH, particularly within the cerebrospinal fluid, are still scarce. Recent evidence suggests the immune response to SAH is dynamic and time-dependent, with some seemingly paradoxical effects reported. An early transient immunosuppression is often observed after aneurysmal SAH. Patients frequently develop lymphopenia in the acute phase, with reductions in circulating T helper cells, cytotoxic T cells, NK cells, and even Tregs [15]. On the other hand, a delayed immune activation appears to occur in the subacute phase. As patients enter the window of vasospasm and DCI typically days 3–7 post-SAH, studies have noted increased immune cell activity in both blood and CSF. In particular, several reports describe an expansion or heightened activation of T lymphocytes during the time when DCI develops [16]. There seems to be an initial phase of immunodepression followed by a phase of immune rebound and immune dysregulation. This complexity makes it all the more crucial to tease apart the roles of specific immune cell subsets in SAH outcomes.

Among the lymphocyte subsets, NK cells have emerged as potential key mediators of injury after SAH. NK cells are innate cytotoxic lymphocytes capable of lysing cells and producing inflammatory cytokines. In SAH patients, NK cell dynamics in the CNS have shown notable changes. Our group reported that NK cells progressively accumulate in the CSF over the first week post-hemorrhage, peaking around day 5–6, before declining [17]. Activated NK cells can inflict direct neurovascular damage by releasing granules containing perforin and granzyme or triggering apoptosis in neurons and endothelial cells. They can secrete pro-inflammatory cytokines like interferon-γ, tumor necrosis factor-α, and the growth factor granulocyte–macrophage colony-stimulating factor that amplify inflammation. These effects may contribute to cerebrovascular spasm and parenchymal injury [15].

In summary, hyperactivation of NK cells in the aftermath of SAH may increase the inflammatory cascade and possibly play a role in the development of DCI. This makes NK cells a compelling target of interest as both a prognostic marker and a possible therapeutic focus. In contrast to NK cells, Tregs are generally viewed as protective, anti-inflammatory players in neuroinflammation [18]. Tregs are a subset of CD4⁺ T cells characterized by FOXP3 expression, and they serve to maintain immune homeostasis and self-tolerance. Most studies investigating Tregs and neuroinflammation have been conducted in patients with ischemic stroke [19–21]. Following SAH, Tregs appear to suppress neuroinflammation, partly through the release of IL-10 [22]. Tregs in peripheral blood were significantly reduced during the early brain injury phase after SAH in patients with cerebral ischemia compared to those without cerebral ischemia [12], and similar findings were reported for Tregs in CSF [23]. Tregs are known to exert powerful modulatory effects on other immune cells: they can suppress the activity of CD4⁺ helper T cells, CD8⁺ cytotoxic T cells, and NK cells through cell-contact mechanisms and the release of anti-inflammatory cytokines [24]. This imbalance, with lower Treg presence in the CSF alongside pro-inflammatory effector T cells, could foster a highly inflammatory milieu in the brain early after SAH. Over time, as blood-derived factors and chemokines alter the blood–brain barrier permeability, more Tregs may infiltrate or become activated in the CNS, potentially contributing to the resolution of inflammation in later stages.

The present study will examine the role of Tregs and cytotoxic NK cells in the context of SAH, with particular focus on their contributions to DCI. By elucidating how these cellular immune responses associate with DCI risk, we aim to advance the understanding of SAH pathophysiology and potentially identify immune biomarkers or therapeutic targets for improving patient outcomes.

## Methods

### Study goal and design

The study investigates an interaction between the activity of CD3⁻ CD16⁺ CD56⁺ CD107a⁺ NK cells and CD3⁺ CD4⁺ CD8⁺/⁻ CD25⁺ FOXP3⁺ Tregs in CSF and blood with DCI following aneurysmal SAH. The study was designed to improve the understanding of immune-cell dynamics after SAH and to generate hypotheses for future biomarker and mechanistic studies.

It adheres to clinical guidelines and standard operating procedures, ensuring patient safety and ethical compliance. The prospective study was performed in accordance with the Declaration of Helsinki as revised in 2013 and its later amendments and was approved by the local ethics committee of the Medical Faculty of the University of Ulm, Germany (Reference Number: 157/20, 23. July 2020). Written informed consent was obtained from all patients or their legal guardians. Patients were recruited from 01.02.2023 to 30.11.2024 at the University Hospital of Ulm, Germany.

CSF and blood samples were collected from patients with aneurysmal SAH up to 14 days post-hemorrhage. Samples were obtained at predetermined time points to evaluate inflammatory and immunoregulatory parameters. Quantification of NK cells and Tregs was conducted using flow cytometry; immunological markers were then analyzed in relation to the occurrence and timing of DCI.

### Patients and clinical management

This study included 21 patients diagnosed with aneurysmal SAH who were admitted to the hospital on the day of the bleeding event and enrolled within a 24-hour period.

Inclusion criteria were:

- Evidence of blood in the CSF, confirmed via computed tomography (CT), magnetic resonance imaging (MRI), or lumbar puncture;
- Radiological confirmation of an intracranial aneurysm as the source of bleeding by CT angiography, MR angiography, or digital subtraction angiography (DSA);
- Presence of an external ventricular drain or lumbar drainage for the management of acute CSF circulation disturbance,
- Age ≥ 18 years; and
- Provision of informed consent by the patient or a legally authorized representative in cases of impaired consciousness.

Exclusion criteria were:

Predicted survival < 48 h, known immunological or oncological disease, or coagulation disorders.

The diagnosis of SAH was confirmed through CT and CT angiography and/or digital subtraction angiography in all cases. The World Federation of Neurosurgical Societies (WFNS) scale [25] was used to grade the clinical condition on admission, and the pattern of hemorrhage on CT was graded using the modified Fisher scale [26].

Treatment strategy, microsurgical versus endovascular aneurysm repair, was jointly determined by a neurosurgeon and a neuroradiologist and performed within 24 h of admission. The external ventricular drain was inserted via frontal ventriculostomy, and the lumbar drain was placed by lumbar puncture under local anesthesia. All patients were subsequently admitted to the intensive care unit (ICU) and received standard care in accordance with current guidelines [27].

Functional outcome following SAH was assessed using modified Rankin Scale (mRS) [28] and Glasgow Outcome Scale (GOS) [29] at hospital discharge and at 3-; 6- and 12-month follow-up after discharge.

### Sampling of CSF and blood specimens

In accordance with the study protocol, 2–4 mL of CSF and 4–8 mL of blood were collected on specific days (d1, d2, d3, d4–6, d7, d8–13, and d14) up to 14 days post-SAH at the University Hospital of Ulm. CSF samples were collected from the clinically indicated CSF drainage device, either an external ventricular drain (EVD) or lumbar drain (LD), under aseptic conditions. The sampling site, ventricular versus lumbar CSF, was recorded for each patient and sampling time point where available. Because ventricular and lumbar CSF may differ in cellular and inflammatory composition, sampling site was considered descriptively where possible; however, the small number of LD samples precluded formal statistical adjustment.

For cell analysis, 2000 µL of CSF per sample was centrifuged at 300 g for 10 min. The CSF supernatant was separated from the cell pellet. For cryopreservation, pellets were resuspended in 10% dimethyl sulfoxide (DMSO, Thermo Fisher Scientific Inc.) and stored in a medium containing 10% DMSO, 10% FBS (FBS, Gibco, Thermo Fisher Scientific Inc.,;? Darmstadt, Germany), and 80% DMEM (DMEM, Thermo Fisher Scientific Inc.).

Whole blood samples were resuspended in cryopreservation medium consisting of 10% DMSO, 10% FBS, and 40% DMEM.

Flow cytometry (FACS) was performed to quantify CD3⁻CD16⁺CD56⁺CD107a⁺ NK cells and CD3⁺CD4⁺CD25⁺FOXP3⁺ Tregs, with CD8 expression used to distinguish CD8⁺ and CD8⁻ subsets.

### Sample preparation and cell staining

Frozen blood and CSF samples were rapidly thawed in a 37 °C water bath until about 90% liquid. Samples were diluted 1:10 in pre-warmed PBS and centrifuged at 200 × g for 10 min at 4 °C. Supernatants were discarded, and cells were resuspended in Fc receptor blocking solution containing Fixable Viability Dye (FVD, Thermo Fisher Scientific Inc.) and incubated for 20 min at 4 °C.

Cells were washed with PBS + 1% bovine serum albumin (BSA, Thermo Fisher Scientific Inc.) and aliquoted for staining with two antibody panels (NK and Treg).

#### NK cell staining (CD3⁻ CD16⁺ CD56⁺ CD107a⁺)

Cells were stained with fluorescent antibodies against CD3 (PerCP), CD16 (APC), CD56 (FITC), and CD107a (PE) (all BioLegend, San Diego, USA). Surface staining was performed for 30 min at 4 °C in the dark. After staining, erythrocytes were lysed with freshly prepared FACS lysing solution (Thermo Fisher Scientific Inc.), and cells were fixed, followed by centrifugation and washing. Cells were resuspended in PBS + 1% BSA for analysis.

#### Regulatory T cell staining (CD3⁺ CD4⁺ CD8⁺/⁻ CD25⁺ FOXP3⁺)

For Treg phenotyping, cells were stained with antibodies against CD3 (PerCP), CD4 (APC), CD8 (APC-Cy7), CD25 (PE), and FoxP3 (AF488) (all BioLegend). Surface staining was performed for 30 min at 4 °C in the dark. Cells were then lysed, fixed, and permeabilized using Cyto-Fast Fix/Perm buffer (Thermo Fisher Scientific Inc.), followed by intracellular FoxP3 staining and washing prior to flow cytometric analysis.

#### Flow cytometric acquisition and analysis

Data acquisition was performed on a BD FACS Lyric (BD Biosciences), collecting up to 500,000 events per sample. Data were analyzed using BD Suite, FlowJo v10.1r5 (Tree Star Inc.), and Cytolution (CytoSolution GmbH, Munich, Germany). NK cells were defined as CD3⁻ CD16⁺ CD56⁺ CD107a⁺, and Tregs as CD3⁺ CD4⁺ CD8⁻ CD25⁺ FOXP3⁺. The NK/Treg ratio was calculated as a dimensionless ratio of NK cells to Tregs for each compartment and sampling time point. Samples with zero detectable Treg counts were excluded from NK/Treg ratio calculations. The detailed flow cytometry gating strategy for NK cells and Tregs is provided in the Supplementary Material.

### Diagnosis and monitoring of delayed cerebral ischemia (DCI)

DCI was defined according to Vergouwen et al. as the occurrence of a new focal neurological deficit (e.g., hemiparesis, aphasia, or neglect) or a decrease in the level of consciousness (a reduction in the Glasgow Coma Scale score of ≥ 2 points lasting for at least 1 h), not immediately after aneurysm occlusion and not attributable to other causes such as rebleeding, hydrocephalus, seizures, metabolic disturbances, or systemic complications; and/or the presence of a cerebral infarction on CT or MRI within 6 weeks after SAH that was not visible on imaging 24–48 h after aneurysm treatment and could not be explained by the initial hemorrhage or procedural complications [30]. Cerebral vasospasm was defined as new or increased arterial narrowing on computed tomography angiography (CTA), magnetic resonance angiography (MRA), or digital subtraction angiography (DSA) compared with baseline imaging, as determined by an experienced neuroradiologist.

Patients underwent daily monitoring of vital parameters, intracranial pressure (ICP) measured via external ventricular drain or intraparenchymal probe, and neurological status evaluated using the Glasgow Coma Scale (GCS) [31].

On day 1, CT and CTA were performed to confirm hemorrhage and identify underlying vascular pathologies, followed by DSA for definitive aneurysm characterization and therapeutic planning. Transcranial Doppler (TCD) examinations were performed daily from day 1 to day 14 to monitor cerebral blood flow velocities in major intracranial arteries. Elevated flow velocities were defined as a mean cerebral blood flow velocity ≥ 120 cm/s or an increase of > 50% within a 24-hour period [32, 33]. These elevated velocities were interpreted as indicators of cerebral vasospasm and as surrogate markers of impending DCI. In such cases, CTA and CT perfusion imaging were subsequently performed to confirm the presence of vasospasm and/or perfusion deficits. TCD monitoring was performed using a Doppler-Box device with standard insonation techniques by trained neurosurgeons. In cases where TCD was not applicable, CTA and CT perfusion imaging were performed on days 3, 7, and 10.

### Statistical analysis and figures

Continuous variables were summarized as mean with standard deviation (SD) or median with range, depending on distribution. Categorical variables were summarized as counts and percentages. Given the small sample size and the non-normal distribution of several immune-cell ratios, between-group comparisons were primarily performed using non-parametric methods. The Mann–Whitney U test was applied to assess differences in NK/Treg ratios between groups defined by DCI status, dichotomized functional outcome, WFNS grade, and modified Fisher grade.

Longitudinal immune-cell dynamics were analyzed to assess temporal changes after SAH. Because repeated measurements were incomplete across time points, mixed-effects models were used where applicable to evaluate the overall effect of time on NK cells, CD107a-positive NK cells, and Tregs. Immune-cell counts were log10-transformed before mixed-effects analysis to reduce skewness. Longitudinal patterns stratified by DCI status were primarily interpreted descriptively because of the limited sample size and high interindividual variability. Statistical significance was defined as *p* ≤ 0.05. Owing to the exploratory nature of this pilot study, p values were interpreted cautiously and no correction for multiple testing was applied. Statistical analyses were performed using GraphPad Prism version 10.6.1 and Microsoft Excel for Mac version 16.78.3. Figures were designed to show individual variability and descriptive temporal patterns. Where bar graphs are retained, mean values and SD are reported.

## Results

### Patient characteristics

A total of 21 patients (16 females, 76.2%) with SAH were included. The mean age was 55.9 years (SD 13.3) and the mean BMI was 27.4 kg/m2 (SD 4.4). The initial clinical condition at admission showed a median WFNS grade of 3 (range 1–5) and a median modified Fisher grade of 3 (range 1–4).

Most aneurysms were located in the anterior circulation (19/21, 90.5%), while two patients (9.5%) had aneurysms in the posterior circulation. Detailed aneurysm locations are provided in Table 1. Six patients (28.6%) presented with multiple aneurysms. CSF was obtained via EVD in 17 patients and via LD in 4 patients. Given the small number of LD samples, no formal statistical adjustment for sampling site was performed.

Endovascular coiling was performed in 12 cases (57.1%), microsurgical clipping in 8 (38.1%), and one patient (4.8%) underwent a combined coiling and clipping procedure.

Radiological vasospasm occurred in 13 patients (61.9%), typically around day 6 (range 3–12) after ictus. DCI developed in 10 patients (47.6%), with a median onset of 8.5 days after hemorrhage (range 4–12). Infectious complications occurred in 9 patients (42.9%), most commonly urinary or respiratory tract infections, while no CSF infections were documented. A ventriculo-peritoneal (VP) shunt was required in 8 patients (38.1%), with one additional delayed shunt insertion (4.8%), more than 30 days after the initial SAH. The mean hospital stay was 24.2 days (SD 9.2).

At discharge, the median Glasgow Outcome Score (GOS) was 4 (range 1–5). Two patients (9.5%) died during hospitalization or follow-up (GOS = 1). At 6 months follow-up, 12 patients (57.1%) achieved a good neurological outcome (GOS 4–5 / mRS 0–2), while 9 patients (42.9%) had a poor outcome (GOS 1–3 / mRS 3–6). Baseline demographic and clinical characteristics of the patients are presented in Table 1.

**Table 1:** Demographic and clinical characteristics

Patient	Age	Gender	BMI	WFNS	mFisher	Aneurysm	Sealing	DCI	Day of DCI
1	70	F	26	5	4	ICA	Clip	Yes	7
2	22	M	21	4	4	ACOM	Coil	No	No
3	50	F	24	4	4	MCA	Coil	Yes	10
4	70	F	25	2	2	PCOM	Coil	Yes	7
5	58	F	28	3	3	MCA	Coil	Yes	4
6	53	F	28	1	1	ICA	Coil	No	No
7	59	F	22	3	3	ACOM	Coil	Yes	8
8	29	F	27	1	2	MCA	Clip	No	No
9	73	M	29	1	3	MCA	Coil	No	No
10	54	F	22	5	4	MCA	Clip	No	No
11	51	F	27	1	3	ACOM	Clip	No	No
12	53	F	31	4	4	ACOM	Coil	No	No
13	69	F	38	3	4	ICA	Coil	No	No
14	63	F	23	2	3	PCOM	Clip	No	No
15	63	F	31	1	3	ACOM	Combined	Yes	8
16	69	M	28	4	3	ACOM	Coil	No	No
17	69	F	29	4	4	ACOM	Clip	Yes	9
18	42	M	32	5	4	MCA	Clip	Yes	9
19	51	F	22	1	3	ICA	Coil	No	No
20	50	F	35	4	4	MCA	Clip	Yes	12
21	55	F	28	4	4	MCA	Coil	Yes	11

Abbreviations: BMI = body mass index; WFNS = World Federation of Neurosurgical Societies scale; mFisher = modified Fisher scale; ICA = internal carotid artery; ACOM = anterior communicating artery; MCA = middle cerebral artery; PCOM = posterior communicating artery; DCI = delayed cerebral ischemia

### FACS analysis

**Fig. 1:**
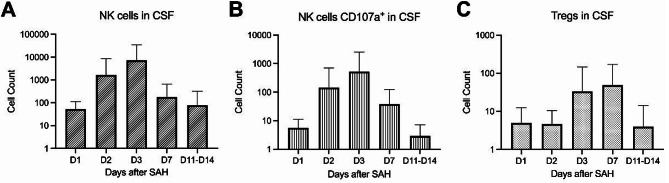
Longitudinal flow cytometry cell counts of NK cells (**A**), cytotoxic NK cells (CD107a-positive) (**B**), and regulatory T cells (**C**) in CSF after SAH. Cell counts are shown across predefined sampling time points. Bars represent mean values and error bars indicate standard deviation. Mixed-effects analysis of log10-transformed counts showed a significant overall effect of time for NK cells (**A**: *p* = 0.0076), CD107a-positive NK cells (**B**: *p* = 0.0008), and Tregs (**C**: *p* = 0.0007)

Flow cytometric analyses showed temporal variation in CSF NK cell counts after SAH (Fig. 1A). NK cell counts increased from day 1 (mean 52.9, SD 58.5) to day 2 (mean 1666.1, SD 6762.1) and were highest at day 3 (mean 7260.2, SD 27858.4). Counts decreased at day 7 (mean 181.1, SD 467.9) and days 11–14 (mean 78.6, SD 242.1). A mixed-effects analysis of log10-transformed counts showed a significant overall effect of time (*n* = 21 patients, 86 observations; χ² (4) = 13.91; *p* = 0.0076).

A comparable temporal pattern was observed for activated NK cells expressing CD107a. Values were low at day 1 (mean 5.6, SD 5.7), increased at day 2 (mean 145.3, SD 563.9) and day 3 (mean 528.2, SD 2022.8), and declined again at day 7 (mean 38.6, SD 85.2) and days 11–14 (mean 2.9, SD 4.3). A mixed-effects analysis of log10-transformed counts showed a significant overall effect of time (*n* = 21 patients, 86 observations; χ² (4) = 18.93; *p* = 0.0008).

In contrast, Tregs were present at low levels at day 1 (mean 4.9, SD 7.6) and day 2 (mean 4.6, SD 5.8), increased at day 3 (mean 33.6, SD 111.7) and day 7 (mean 48.9, SD 121.8), and declined again by days 11–14 (mean 4.0, SD 10.1). A mixed-effects analysis of log10-transformed Treg counts showed a significant overall effect of time (*n* = 21 patients, 86 observations; χ² (4) = 19.22; *p* = 0.0007).

**Fig. 2:**
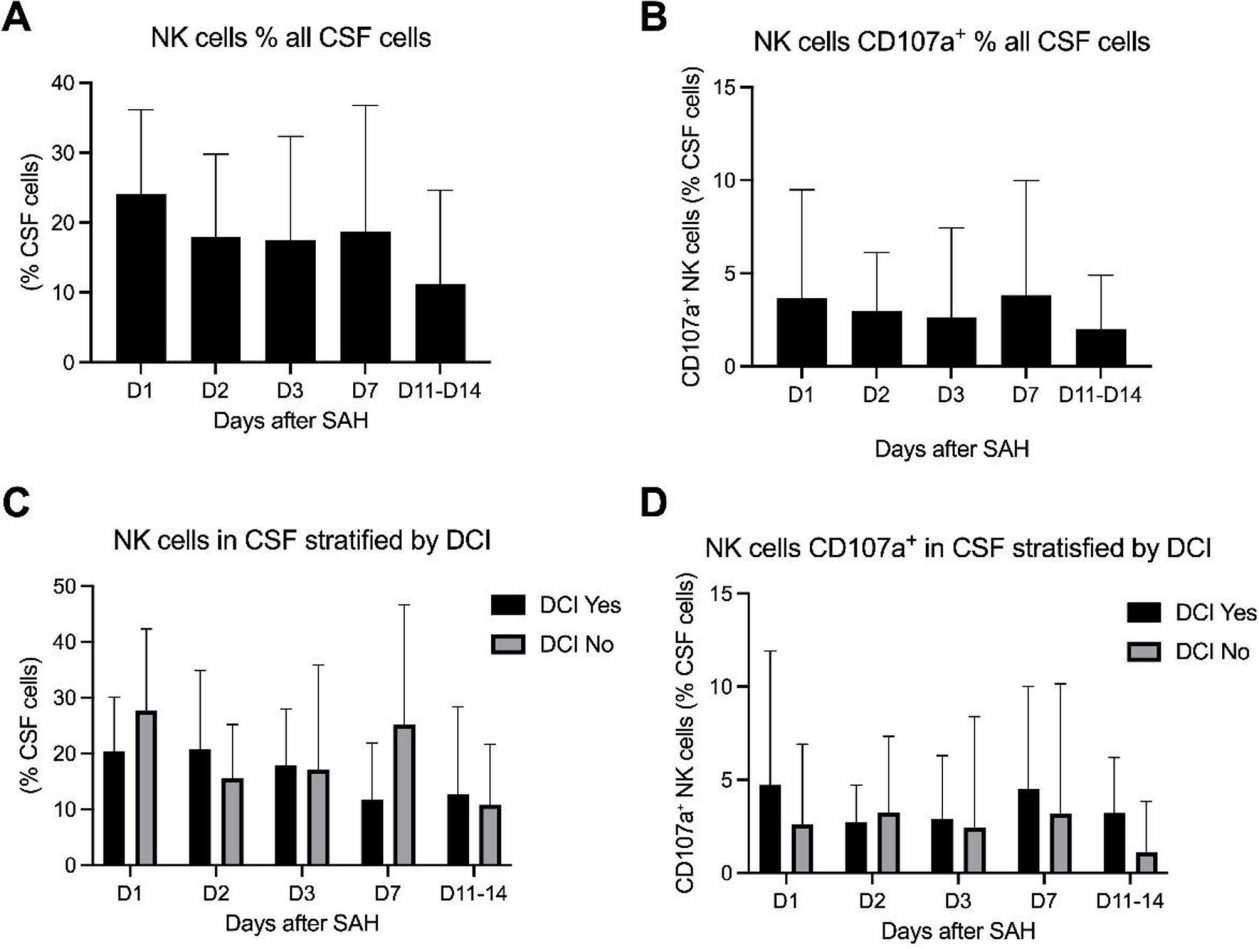
Longitudinal dynamics of NK cells and CD107a⁺ NK cells in CSF between day 1 and day 14. Data are shown for the overall cohort (**A**, **B**) and stratified by DCI status (**C**, **D**). Values are presented as mean and standard deviation

In the CSF, NK cell proportions differed only modestly between patients with and without DCI. At day 3, patients who subsequently developed DCI exhibited a slightly higher mean NK fraction compared with patients without DCI (17.9% vs. 17.1%). By day 7, NK cell proportions declined descriptively in the DCI group, whereas they increased in patients without DCI (25.1%). Between-group comparisons at individual time points were performed using the Mann–Whitney U test and did not show statistically significant differences in CSF NK cell proportions between patients with and without DCI. These findings are illustrated in Fig. 2, showing longitudinal NK cell dynamics in CSF in the overall cohort and stratified by DCI status.

In addition to proportional differences, NK cell activation in the CSF varied markedly between individuals. This was assessed by CD107a mean fluorescence intensity, normalized to the total number of CSF cells. Three distinct activation patterns were observed across the cohort, including persistently low or undetectable activation, moderate and stable activation, and marked hyperactivation characterized by transient or sustained peak values. When stratified by DCI status, patients with DCI tended to show higher and more dynamic CD107a expression, with repeated or sustained peaks occurring predominantly in this group. In contrast, a persistently low or inactive activation pattern was observed exclusively in patients without DCI. Although peak CD107a values were numerically higher in patients with DCI, these differences did not reach statistical significance, and the association between a low activation phenotype and absence of DCI remained a non-significant trend.

**Fig. 3:**
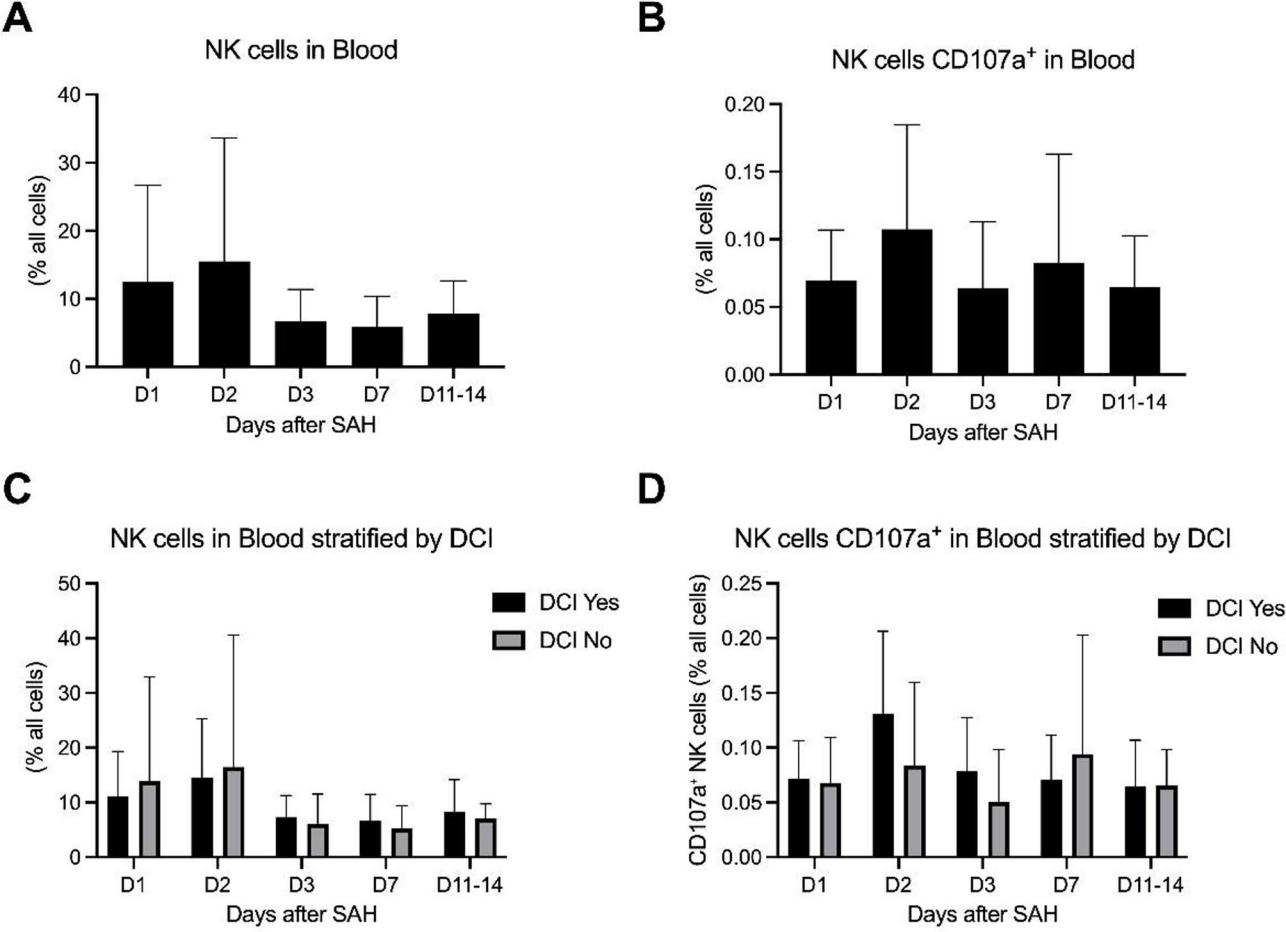
Longitudinal dynamics of NK cells and CD107a⁺ NK cells in blood between day 1 and day 14. Data are shown for the overall cohort (**A**, **B**) and stratified by DCI status (**C**, **D**). Values are presented as mean and standard deviation

In peripheral blood, NK cell proportions followed a broadly similar temporal pattern but remained consistently lower than in CSF. At day 3, NK fractions were slightly higher in patients with DCI than in patients without DCI (7.3% vs. 6.1%), and this small numerical difference persisted at day 7 (6.6% vs. 5.2%). However, these between-group differences did not reach statistical significance. Correspondingly, peripheral blood NK cell activation, assessed by CD107a mean fluorescence intensity, showed low overall levels with limited variability across time points and no statistically significant differences between patients with and without DCI. Occasional isolated peak values were observed in both groups, but lacked a consistent temporal or group-specific pattern. These longitudinal dynamics are shown in Fig. 3, which illustrates NK cell proportions and CD107a-positive NK cell activation in blood over time for the overall cohort and stratified by DCI status.

**Fig. 4:**
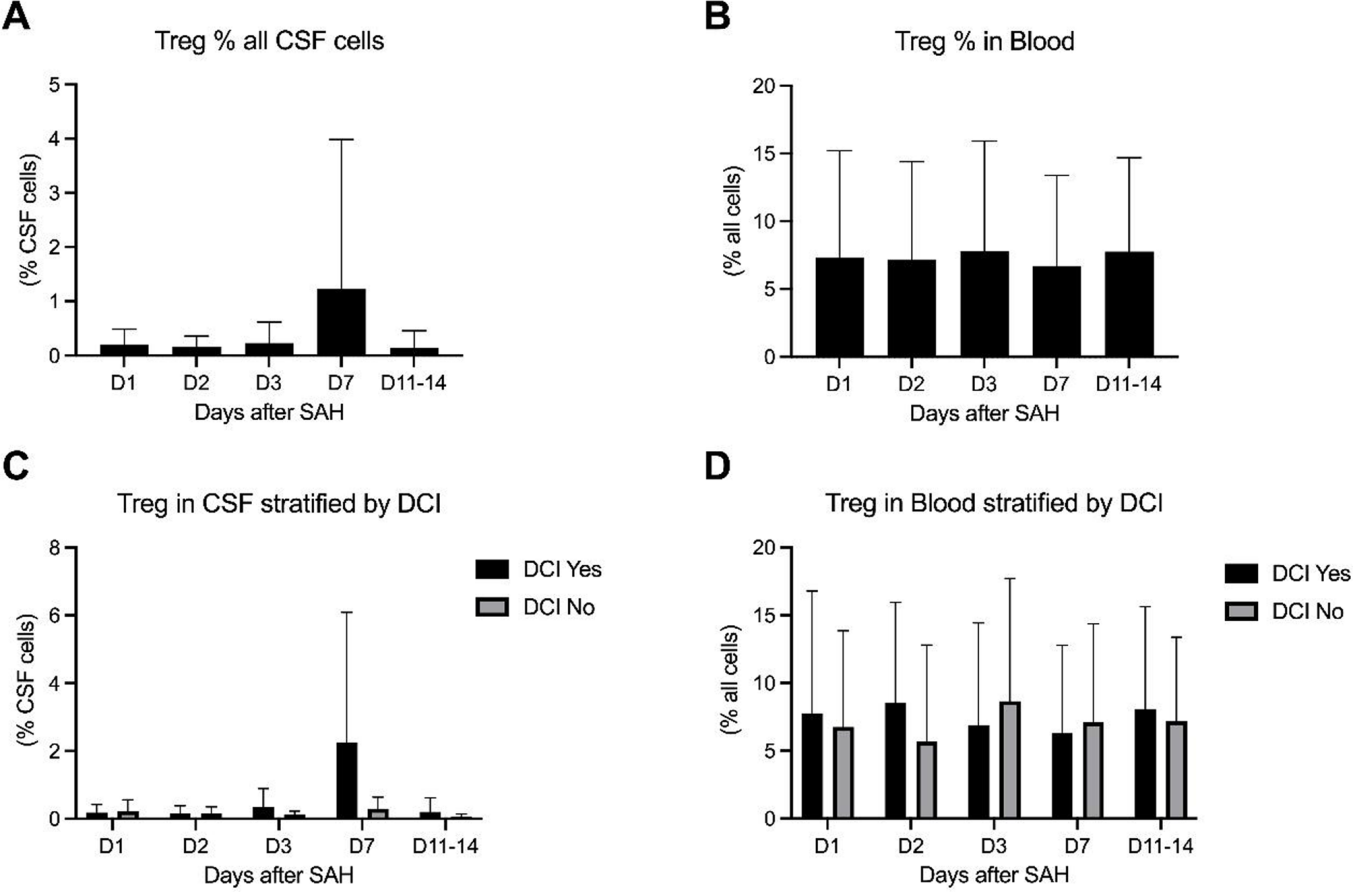
Longitudinal dynamics of regulatory T cells between day 1 and day 14 after SAH. Data are shown for CSF and peripheral blood in the overall cohort (**A**, **B**) and stratified by DCI status for the respective compartments (**C**, **D**). Values are presented as mean and standard deviation

Tregs in CSF remained consistently low, but with notable group differences at later timepoints. At day 3, DCI patients exhibited higher Treg levels (0.4%) compared with non-DCI patients (0.2%). By day 7, this divergence became more pronounced, with mean Treg levels reaching 2.9% in the DCI group versus 0.4% in non-DCI patients, suggesting a delayed intrathecal regulatory response in DCI, see Fig. 4.

In blood, Treg dynamics differed from CSF. At day 3, non-DCI patients displayed higher circulating Treg fractions (8.7%) compared to DCI patients (6.9%). By day 7, the difference narrowed (6.3% in DCI vs. 7.1% in non-DCI), reflecting partial convergence of systemic regulatory responses across groups. Altogether the difference was mild and statistically not significant.

**Fig. 5:**
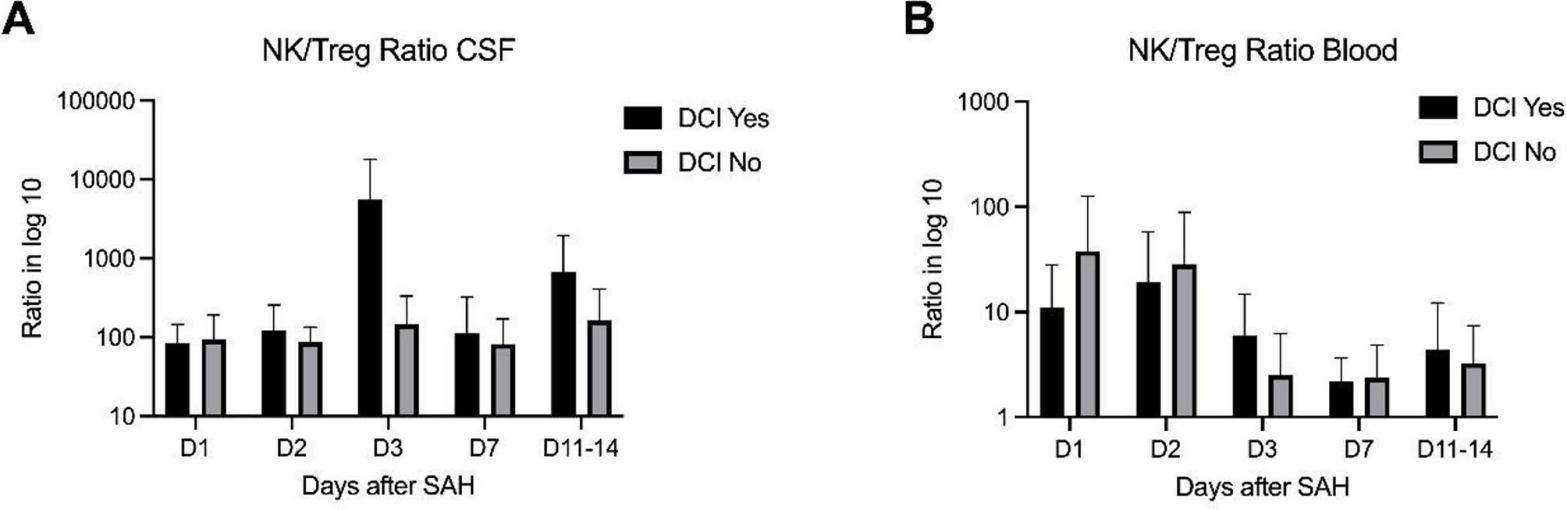
Longitudinal changes in the NK/Treg ratio in CSF (**A**) and blood (**B**) between day 1 and day 14, stratified by the presence of DCI. Values are displayed on a log₁₀ scale and presented as mean and standard deviation

The resulting NK/Treg ratios underscored an imbalance, particularly in CSF. At day 3, the NK/Treg ratio was markedly higher in DCI patients (5556.6 SD 12266.0) compared with non-DCI patients (161.8 SD 195.2). Although the ratio decreased by day 7, it remained elevated in the DCI group (111.5 SD 209.6), compared with the non-DCI group (73.9 SD 87.9). In blood, ratios were markedly lower than in CSF. At day 3, patients with DCI exhibited a higher NK/Treg ratio (6.0 SD 8.6) than those without DCI (2.5 SD 3.7), whereas by day 7 values were comparable between groups (2.2 SD 1.4 in DCI and 2.8 SD 2.6 in non-DCI). Comparisons stratified by DCI status did not reach statistical significance. The corresponding graphs are shown in Fig. 5.

### Clinical and radiological parameters

In exploratory analyses of clinical and radiological covariates, patients with lower modified Fisher grades (1–3) showed higher CSF NK/Treg ratios than patients with modified Fisher grade 4 on day 1 and day 2 after SAH. On day 1, the mean CSF NK/Treg ratio was 113.1 (SD 71.8) in patients with modified Fisher grades 1–3 compared with 14.5 (SD 7.2) in patients with modified Fisher grade 4 (Mann–Whitney U test, *p* = 0.046). On day 2, the mean CSF NK/Treg ratio was 153.7 (SD 100.8) in patients with modified Fisher grades 1–3 compared with 35.1 (SD 25.6) in patients with modified Fisher grade 4 (Mann–Whitney U test, *p* = 0.007). In contrast, no statistically significant associations were observed between NK/Treg ratios and DCI occurrence, 6-month functional outcome, or initial WFNS grade in dichotomized group comparisons using the Mann–Whitney U test.

## Discussion

Taken together, these findings indicate that patients who develop DCI exhibit an early imbalance in intrathecal immunity, with relatively preserved NK cell fractions but disproportionately low Treg representation, leading to extremely high NK/Treg ratios in CSF. In contrast, systemic immune changes were comparatively subtle and showed partial normalization over time. The observed interaction of early intrathecal NK/Treg ratios with modified Fisher grade further suggests that the extent of subarachnoid blood load contributes to the magnitude of early inflammatory dysregulation within the CSF compartment. However, given the small cohort, high interindividual variability, and limited number of statistically significant between-group findings, these results should be interpreted with appropriate caution.

Among all patients enrolled in our study, 10 (47.6%) developed DCI during their hospital course. DCI occurred with a median onset of 8.5 days after hemorrhage (range 4–12), aligning with prior observations on the typical timing of DCI in this population [34]. In the overall cohort, CSF immune-cell counts changed significantly over time after SAH. Mixed-effects analysis of log10-transformed counts showed a significant overall effect of time for NK cells, CD107a-positive NK cells, and Tregs. This supports the presence of a dynamic intrathecal immune response during the early phase after SAH, although the high interindividual variability warrants cautious interpretation.

In CSF, immune cell dynamics diverged early between patients who developed DCI and those who did not. By day 3 after SAH, patients who later developed DCI showed a sharp increase in NK cells with persistently low Treg counts, resulting in a markedly elevated NK/Treg ratio compared with non-DCI patients (mean 5556.6 vs. 162.8). This imbalance persisted at day 7 (mean 111.5 vs. 73.9) and remained detectable at later time points (days 11–14; mean 672.8 vs. 148.4), indicating a sustained intrathecal predominance of NK-cell–driven inflammation in patients who developed DCI. These data are consistent with the hypothesis that an early increase in intrathecal NK cells together with relatively low Treg representation may precede the development of DCI.

Peripheral blood samples demonstrated similar trends of NK cell and Treg dynamics, but the changes were considerably less pronounced than in the CSF. We noted a mild early increase in circulating NK cell levels after SAH and a concomitant tendency for Treg counts in blood to decline, especially in patients who eventually developed DCI. This led to an elevated NK/Treg ratio in blood at day 3 in the DCI group (mean 5.96) relative to the non-DCI group (2.53). However, by day 7, systemic NK and Treg levels had begun to converge between the two groups. Overall, the immune alterations associated with DCI were far more accentuated in the CSF compartment than in peripheral blood. This compartmentalization underscores that the inflammatory response to SAH, particularly the surge in cytotoxic NK cells and the relative paucity of Treg cells, is largely a central phenomenon in those who develop DCI. Consistently, in a previous study we have reported that NK cell activation in the CSF correlates with cerebrovascular complications after SAH [17]. We observed that activated cytotoxic NK cells in CSF peak around the time of DCI onset and concluded that an early increase in NK cells is associated with higher risk of DCI. Our findings are in line with this report: patients with an early, robust intrathecal NK cell response and concurrently low Treg counts were those who suffered subsequent DCI, suggesting that immune monitoring in the CSF could provide an early warning for impending DCI.

Given that NK cells are considerably more common than regulatory T cells in peripheral blood, a purely blood-burden–driven effect would be expected to result in higher CSF NK/Treg ratios with increasing modified Fisher grades [35, 36]. However, this pattern was not observed in our data. Instead, lower modified Fisher grades were associated with higher intrathecal NK/Treg ratios at early time points. Although this finding should be interpreted cautiously, it argues against passive blood contamination as the sole explanation for the observed CSF immune profile. Together with the lack of a comparable association in peripheral blood, these results suggest that early CSF NK/Treg dynamics after SAH may at least partly reflect compartment-specific immune processes rather than hemorrhage burden alone.

### Mechanistic implications

NK cells are potent producers of interferon‑γ and tumor‑necrosis‑factor-α (TNF‑α) [37]. Both cytokines can aggravate endothelial dysfunction, promote cerebral‑artery vasospasm and amplify blood‑brain‑barrier (BBB) disruption after SAH [6, 38]. In contrast, Tregs exert neuroprotective effects by suppressing effector‑T‑cell proliferation, limiting neutrophil infiltration and secreting anti‑inflammatory mediators such as IL‑10 and transforming growth factor‑β [19, 39].

Experimental SAH models support this dichotomy. Low‑dose IL‑2, which selectively expands Tregs, reduces matrix‑metalloproteinase‑9-mediated BBB breakdown, attenuates cerebral edema and improves neurological outcome in rats. Moreover, adoptive transfer of Tregs diminishes vasospasm severity and improves cerebral perfusion in a mouse SAH model [40]. Together, these experimental studies provide biological plausibility for the hypothesis that an altered balance between cytotoxic and regulatory immune mechanisms may contribute to secondary injury after SAH. However, the present clinical study cannot determine whether the observed NK/Treg ratio is a causal driver of DCI, a consequence of evolving injury, or an epiphenomenon of broader neuroinflammatory activation.

### Clinical relevance

Our findings suggest that CSF immune profiling during the first week after SAH may provide exploratory information on immune dynamics associated with high DCI risk. Higher CSF NK/Treg ratios on day 3 were observed in patients who later developed DCI; however, the present cohort is too small to define or validate a clinically applicable threshold. The comparatively modest changes in peripheral blood suggest that systemic biomarkers may not fully reflect intrathecal immune alterations after SAH, supporting further evaluation of CSF-based immune profiling in larger prospective studies.

NK‑cell dynamics after ischemic stroke are marked by an accumulation of NK cells within the brain parenchyma, whereas peripheral blood NK‑cell counts tend to decline modestly but often fail to reach statistical significance [41]. This pattern is in line with our findings of compartment-specific NK-cell dynamics and highlights the importance of considering the NK/Treg balance across CSF and blood.

In a mouse model, Tregs infiltrated into the brain one day after SAH and exerted neuroprotective effects by secreting IL-10 to suppress neuroinflammation and reduce neuron apoptosis [22]. Using a rat SAH model, Dong et al. reported a marked reduction in circulating regulatory T‑cells after SAH, which began to recover by day 3. The authors then examined the neuroprotective potential of low‑dose interleukin‑2 (IL-2). IL‑2 administration restored peripheral Treg numbers and significantly attenuated neuronal injury [42]. Adoptive transfer of Tregs reduced SAH‑induced brain edema and enhanced cerebral blood flow in a rat model. This neurovascular protection was accompanied by attenuation of cerebral inflammation via suppression of the Toll‑like receptor‑4 and NF‑κB signaling pathways [43].

Together, these findings underscore the relevance of immune regulation in secondary brain injury after SAH and suggest that Tregs may contribute to limiting neuroinflammatory cascades implicated in DCI. Future clinical studies will be required to clarify the relevance of this immune crosstalk in patients and to evaluate whether targeted immunomodulation can reduce the incidence or severity of DCI.

### Limitations and future directions

This study, as a pilot study, must be interpreted in light of several important limitations. The cohort was small (*n* = 21) and derived from a single tertiary care center, which limits statistical power and the generalizability of the findings. Moreover, the clinical course of SAH in this series was highly heterogeneous, ranging from severe delayed cerebral ischemia to both favorable and unfavorable clinical outcomes. Most between-group differences in immune-cell dynamics did not reach statistical significance, and several observations were based on numerical trends only. Accordingly, the results should be considered hypothesis-generating rather than conclusive.

Another important limitation is the CSF sampling site. CSF was obtained through either an EVD or an LD, depending on clinical indication. Ventricular and lumbar CSF may differ in cellular composition and inflammatory profile, and the small number of LD samples precluded meaningful adjustment for sampling site. Therefore, sampling location may have influenced the observed immune-cell ratios.

Because the study was observational, immune-cell counts were obtained at predefined intervals rather than continuously, and no experimental manipulation of the immune response was performed. Consequently, causality cannot be established, and the observed NK/Treg imbalance may reflect a downstream epiphenomenon of a broader inflammatory cascade rather than a primary driver of delayed ischemia. Finally, we did not assess genetic or epigenetic factors that could modulate individual immune responses and thereby influence the NK/Treg ratio.

Taken together, these constraints highlight the need for prospective, multicenter studies with larger sample sizes, standardized CSF-sampling protocols, and comprehensive panels of systemic and intrathecal biomarkers, together with careful assessment of concurrent therapies. Integrating CSF immune profiling with established radiological parameters, such as CT angiography and perfusion imaging, and clinical risk scores, such as Hunt-Hess and Fisher grades, may ultimately enable a more precise and personalized treatment approach for patients with aneurysmal SAH.

In summary, an early and sustained intrathecal predominance of NK cells over regulatory T cells was observed in patients who developed DCI after aneurysmal SAH. This imbalance may represent a pathophysiologically relevant component of secondary ischemic injury and could provide a basis for future studies on prognostic assessment and therapeutic intervention. To our knowledge, this is the first study to investigate the NK/Treg ratio in this context.

## Supplementary Information

Below is the link to the electronic supplementary material.

Supplementary Material 1

## Data Availability

No datasets were generated or analysed during the current study.

## References

[1] Ziebart A, Dremel J, Hetjens S, Nieuwkamp DJ, Linn FH, Etminan N, et al. Case fatality and functional outcome after spontaneous subarachnoid haemorrhage: A systematic review and meta-analysis of time trends and regional variations in population-based studies. Eur Stroke J. 2024;9(3):555–65. 38353205 10.1177/23969873241232823 PMC11418425

[2] Veldeman M, Rossmann T, Haeren R, Vossen LV, Weiss M, Conzen C, et al. Delayed Cerebral Infarction After Aneurysmal Subarachnoid Hemorrhage: Location, Distribution Patterns, Infarct Load, and Effect on Outcome. Neurology. 2024;103(3):e209607. 38950352 10.1212/WNL.0000000000209607

[3] Rowland MJ, Hadjipavlou G, Kelly M, Westbrook J, Pattinson KT. Delayed cerebral ischaemia after subarachnoid haemorrhage: looking beyond vasospasm. Br J Anaesth. 2012;109(3):315–29. 22879655 10.1093/bja/aes264

[4] Suzuki H, Kanamaru H, Kawakita F, Asada R, Fujimoto M, Shiba M. Cerebrovascular pathophysiology of delayed cerebral ischemia after aneurysmal subarachnoid hemorrhage. Histol Histopathol. 2021;36(2):143–58. 32996580 10.14670/HH-18-253

[5] Thilak S, Brown P, Whitehouse T, Gautam N, Lawrence E, Ahmed Z, et al. Diagnosis and management of subarachnoid haemorrhage. Nat Commun. 2024;15(1):1850. 38424037 10.1038/s41467-024-46015-2 PMC10904840

[6] Fu X-M, Li C-L, Jiang H-R, Zhang J-y, Sun T, Zhou F. Neuroinflammatory response after subarachnoid hemorrhage: A review of possible treatment targets. Clin Neurol Neurosurg. 2025;252:108843. 40107192 10.1016/j.clineuro.2025.108843

[7] Romoli M, Giammello F, Mosconi MG, De Mase A, De Marco G, Digiovanni A, et al. Immunological profile of vasospasm after subarachnoid hemorrhage. Int J Mol Sci. 2023;24(10). 10.3390/ijms24108856 PMC10218712 37240207

[8] Roa JA, Sarkar D, Zanaty M, Ishii D, Lu Y, Karandikar NJ, et al. Preliminary results in the analysis of the immune response after aneurysmal subarachnoid hemorrhage. Sci Rep. 2020;10(1):11809. 32678268 10.1038/s41598-020-68861-y PMC7367262

[9] Pfnür A, Mayer B, Dörfer L, Tumani H, Spitzer D, Huber-Lang M, et al. Regulatory T Cell- and Natural Killer Cell-Mediated Inflammation, Cerebral Vasospasm, and Delayed Cerebral Ischemia in Aneurysmal Subarachnoid Hemorrhage—A Systematic Review and Meta-Analysis Approach. Int J Mol Sci. 2025;26(3):1276. 39941044 10.3390/ijms26031276 PMC11818301

[10] Provencio JJ, Fu X, Siu A, Rasmussen PA, Hazen SL, Ransohoff RM. CSF neutrophils are implicated in the development of vasospasm in subarachnoid hemorrhage. Neurocrit Care. 2010;12(2):244–51. 19967568 10.1007/s12028-009-9308-7 PMC2844469

[11] Mohme M, Sauvigny T, Mader MM, Schweingruber N, Maire CL, Runger A, et al. Immune Characterization in Aneurysmal Subarachnoid Hemorrhage Reveals Distinct Monocytic Activation and Chemokine Patterns. Transl Stroke Res. 2020;11(6):1348–61. 31858408 10.1007/s12975-019-00764-1

[12] Chaudhry SR, Kahlert UD, Kinfe TM, Endl E, Dolf A, Niemelä M, et al. Differential polarization and activation dynamics of systemic T helper cell subsets after aneurysmal subarachnoid hemorrhage (SAH) and during post-SAH complications. Sci Rep. 2021;11(1):14226. 34244562 10.1038/s41598-021-92873-x PMC8270974

[13] Gan Y, Liu Q, Wu W, Yin JX, Bai XF, Shen R, et al. Ischemic neurons recruit natural killer cells that accelerate brain infarction. Proc Natl Acad Sci USA. 2014;111(7):2704-9. 10.1073/pnas.1315943111 PMC3932858 24550298

[14] Wang H, Wang Z, Wu Q, Yuan Y, Cao W, Zhang X. Regulatory T cells in ischemic stroke. CNS Neurosci Ther. 2021;27(6):643–51. 33470530 10.1111/cns.13611 PMC8111493

[15] Jin J, Duan J, Du L, Xing W, Peng X, Zhao Q. Inflammation and immune cell abnormalities in intracranial aneurysm subarachnoid hemorrhage (SAH): Relevant signaling pathways and therapeutic strategies. Front Immunol. 2022;13:1027756. 36505409 10.3389/fimmu.2022.1027756 PMC9727248

[16] Chaudhry SR, Kahlert UD, Kinfe TM, Endl E, Dolf A, Niemela M, et al. Differential polarization and activation dynamics of systemic T helper cell subsets after aneurysmal subarachnoid hemorrhage (SAH) and during post-SAH complications. Sci Rep. 2021;11(1):14226. 34244562 10.1038/s41598-021-92873-x PMC8270974

[17] Spitzer D, Spitzer NJ, Deininger M, Wirtz CR, Konig R, Burster T, et al. Activation of Cytotoxic Natural Killer Cells After Aneurysmal Subarachnoid Hemorrhage. World Neurosurg. 2017;101:666–76. e1. 28323187 10.1016/j.wneu.2017.03.026

[18] Liesz A, Suri-Payer E, Veltkamp C, Doerr H, Sommer C, Rivest S, et al. Regulatory T cells are key cerebroprotective immunomodulators in acute experimental stroke. Nat Med. 2009;15(2):192–9. 19169263 10.1038/nm.1927

[19] Xu Z, Xu Z, Lu J, Zhang J, Shi L. Regulatory T cells as novel cell-based therapy for ischemic stroke. J Cereb Blood Flow Metab. 2025;45(10):1859–76. 40801337 10.1177/0271678X251366071 PMC12350315

[20] Hurst DA, Sohrabji F. Interleukin-2 Mediated Expansion of T-Regulatory Cells as an Ischemic Stroke Therapy. Stroke. 2024;55(6):e159–60. 38787931 10.1161/STROKEAHA.124.047357

[21] Zhang Y, Liesz A, Li P. Coming to the Rescue: Regulatory T Cells for Promoting Recovery After Ischemic Stroke. Stroke. 2021;52(12):e837–41. 34807742 10.1161/STROKEAHA.121.036072

[22] Zhou J, Yang F, Li H, Xu P, Wang Z, Shao F, et al. Regulatory T cells secrete IL10 to suppress neuroinflammation in early stage after subarachnoid hemorrhage. Med (Kaunas). 2023;59(7). 10.3390/medicina59071317 PMC10383056 37512128

[23] Moraes L, Trias N, Brugnini A, Grille P, Lens D, Biestro A, et al. TH17/Treg imbalance and IL-17A increase after severe aneurysmal subarachnoid hemorrhage. J Neuroimmunol. 2020;346. 10.1016/j.jneuroim.2020.577310 32623101

[24] Cava AL, Kaer LV, Fu Dong S. CD4 + CD25+ Tregs and NKT cells: regulators regulating regulators. Trends Immunol. 2006;27(7):322–7. 16735139 10.1016/j.it.2006.05.003

[25] Report of World Federation of Neurological Surgeons Committee on a universal subarachnoid hemorrhage grading scale. J Neurosurg. 1988;68(6). 10.3171/jns.1988.68.6.0985 3131498

[26] Frontera JA, Claassen J, Schmidt JM, Wartenberg KE, Temes R, Connolly ES Jr., et al. Prediction of symptomatic vasospasm after subarachnoid hemorrhage: the modified fisher scale. Neurosurgery. 2006;59(1):21–7. discussion – 7. 16823296 10.1227/01.neu.0000243277.86222.6c

[27] Treggiari MM, Rabinstein AA, Busl KM, Caylor MM, Citerio G, Deem S, et al. Guidelines for the Neurocritical Care Management of Aneurysmal Subarachnoid Hemorrhage. Neurocrit Care. 2023;39(1):1–28. 37202712 10.1007/s12028-023-01713-5

[28] van Swieten JC, Koudstaal PJ, Visser MC, Schouten HJ, van Gijn J. Interobserver agreement for the assessment of handicap in stroke patients. Stroke. 1988;19(5):604–7. 3363593 10.1161/01.str.19.5.604

[29] Jennett B, Bond M. Assessment of outcome after severe brain damage. Lancet. 1975;1(7905):480–4. 46957 10.1016/s0140-6736(75)92830-5

[30] Vergouwen MD, Vermeulen M, van Gijn J, Rinkel GJ, Wijdicks EF, Muizelaar JP, et al. Definition of delayed cerebral ischemia after aneurysmal subarachnoid hemorrhage as an outcome event in clinical trials and observational studies: proposal of a multidisciplinary research group. Stroke. 2010;41(10):2391–5. 20798370 10.1161/STROKEAHA.110.589275

[31] Teasdale G, Jennett B. Assessment of coma and impaired consciousness. A practical scale. Lancet. 1974;2(7872):81–4. 4136544 10.1016/s0140-6736(74)91639-0

[32] Vora YY, Suarez-Almazor M, Steinke DE, Martin ML, Findlay JM. Role of Transcranial Doppler Monitoring in the Diagnosis of Cerebral Vasospasm after Subarachnoid Hemorrhage. Neurosurgery. 1999;44(6):1237–47. 10371622

[33] Simm RF, de Aguiar PHP, de Oliveira Lima M, Paiva BL. Transcranial Doppler as a Routine in the Treatment of Vasospasm Following Subarachanoid Hemorrhage (SAH). In: Zuccarello M, Clark JF, Pyne-Geithman G, Andaluz N, Hartings JA, Adeoye OM, editors. Cerebral Vasospasm: Neurovascular Events After Subarachnoid Hemorrhage. Vienna: Springer Vienna; 2013. pp. 75–6. 10.1007/978-3-7091-1192-5_16 22890648

[34] Schmidt TP, Weiss M, Hoellig A, Nikoubashman O, Schulze-Steinen H, Albanna W, et al. Revisiting the Timeline of Delayed Cerebral Ischemia After Aneurysmal Subarachnoid Hemorrhage: Toward a Temporal Risk Profile. Neurocrit Care. 2022;37(3):735–43. 35790670 10.1007/s12028-022-01545-9 PMC9672023

[35] Fontenot JD, Gavin MA, Rudensky AY. Foxp3 programs the development and function of CD4 + CD25+ regulatory T cells. Nat Immunol. 2003;4(4):330–6. 12612578 10.1038/ni904

[36] Freud AG, Mundy-Bosse BL, Yu J, Caligiuri MA. The Broad Spectrum of Human Natural Killer Cell Diversity. Immunity. 2017;47(5):820–33. 29166586 10.1016/j.immuni.2017.10.008 PMC5728700

[37] Wang R, Jaw JJ, Stutzman NC, Zou Z, Sun PD. Natural killer cell-produced IFN-γ and TNF-α induce target cell cytolysis through up-regulation of ICAM-1. J Leukoc Biol. 2012;91(2):299–309. 22045868 10.1189/jlb.0611308 PMC3290424

[38] Duan M, Xu Y, Li Y, Feng H, Chen Y. Targeting brain-peripheral immune responses for secondary brain injury after ischemic and hemorrhagic stroke. J Neuroinflammation. 2024;21(1):102. 38637850 10.1186/s12974-024-03101-y PMC11025216

[39] Gendelman HE, Appel SH. Neuroprotective activities of regulatory T cells. Trends Mol Med. 2011;17(12):687–8. 21996344 10.1016/j.molmed.2011.08.005 PMC5892451

[40] Sun H, Lee HS, Kim SH, Fernandes de Lima M, Gingras AR, Du Q, et al. IL-2 can signal via chemokine receptors to promote regulatory T cells’ suppressive function. Cell Rep. 2023;42(8):112996. 37598341 10.1016/j.celrep.2023.112996 PMC10564087

[41] Chen C, Ai Q-D, Chu S-F, Zhang Z, Chen N-H. NK cells in cerebral ischemia. Biomed Pharmacother. 2019;109:547–54. 30399590 10.1016/j.biopha.2018.10.103

[42] Dong G, Li C, Hu Q, Wang Y, Sun J, Gao F, et al. Low-Dose IL-2 Treatment Affords Protection against Subarachnoid Hemorrhage Injury by Expanding Peripheral Regulatory T Cells. ACS Chem Neurosci. 2021;12(3):430–40. 33476129 10.1021/acschemneuro.0c00611

[43] Wang Y, Mao L, Zhang L, Zhang L, Yang M, Zhang Z, et al. Adoptive Regulatory T-cell Therapy Attenuates Subarachnoid Hemor-rhage-induced Cerebral Inflammation by Suppressing TLR4/NF-B Signaling Pathway. Curr Neurovasc Res. 2016;13(2):121–6. 26972078 10.2174/1567202613666160314151536

