# Supplementary material for: Natural killer cells and regulatory T cells in aneurysmal subarachnoid hemorrhage in peripheral blood and cerebrospinal fluid - a pilot study
Source: Fluids Barriers CNS. 2026 May 29;23:77. doi: 10.1186/s12987-026-00824-3 (PMC13227750; doi:10.1186/s12987-026-00824-3)
Supplement: Supplementary file 1 — Supplementary Material 1 [file 12987_2026_824_MOESM1_ESM.docx]

Flow cytometric gating strategy for NK cells. After exclusion of doublets, lymphocytes were identified by their forward- and side-scatter characteristics. Viable cells were then selected, followed by gating on CD3⁻ cells to exclude T cells. NK cells were subsequently defined as CD56⁺ CD16⁺ cells within the viable CD3⁻ lymphocyte population. Degranulation activity was quantified by CD107a mean fluorescence intensity.

Flow cytometric gating strategy for Tregs. After exclusion of doublets, lymphocytes were identified by forward- and side-scatter properties and viable cells were selected. CD3⁺ T cells were then gated and further restricted to CD4⁺ cells. Regulatory T cells were defined as CD25⁺ FoxP3⁺ cells within the CD4⁺ T-cell population.
